# Cyanobacteria and Cyanotoxins: From Impacts on Aquatic Ecosystems and Human Health to Anticarcinogenic Effects

**DOI:** 10.3390/toxins5101896

**Published:** 2013-10-23

**Authors:** Giliane Zanchett, Eduardo C. Oliveira-Filho

**Affiliations:** Universitary Center of Brasilia—UniCEUB—SEPN 707/907, Asa Norte, Brasília, CEP 70790-075, Brasília, Brazil; E-Mail: gilianezanchett@gmail.com

**Keywords:** cyanobacteria, proliferation, cyanotoxins, toxicity, cancer

## Abstract

Cyanobacteria or blue-green algae are among the pioneer organisms of planet Earth. They developed an efficient photosynthetic capacity and played a significant role in the evolution of the early atmosphere. Essential for the development and evolution of species, they proliferate easily in aquatic environments, primarily due to human activities. Eutrophic environments are conducive to the appearance of cyanobacterial blooms that not only affect water quality, but also produce highly toxic metabolites. Poisoning and serious chronic effects in humans, such as cancer, have been described. On the other hand, many cyanobacterial genera have been studied for their toxins with anticancer potential in human cell lines, generating promising results for future research toward controlling human adenocarcinomas. This review presents the knowledge that has evolved on the topic of toxins produced by cyanobacteria, ranging from their negative impacts to their benefits.

## 1. Introduction

Cyanobacteria or blue green algae are prokaryote photosynthetic organisms and feature among the pioneering organisms of planet Earth. They have been in existence for at least 2.7 billion years, and are considered to be the main primary producers of organic matter and the first organisms to have released oxygen into the primitive atmosphere [1]. 

An interesting characteristic of cyanobacteria is their broad geographical distribution, which reflects the group’s genotypic and phenotypic variation [2]. They can be found in limnic and marine environments, but many species are capable of living in terrestrial habitats, where they are important in ecosystem processes and in nutrient cycling. Other species are capable of prospering under environmental stress, such as the extreme environments of Antarctica or thermal springs, or even surviving for long periods in complete darkness [3,4]. Coral reefs, especially degraded ones, are an example of the type of habitat where cyanobacteria are increasingly dominant, due to their capacity for tolerating certain environmental conditions associated with anthropogenic impacts and climate change [5].

This group of organisms manages to tolerate huge changes in salinity and temperature, and their ability also to survive low light intensity gives them an extremely large competitive advantage in these environments [3,6]. Cyanobacteria can multiply quickly in surface waters and form blooms when favorable conditions prevail, such as high temperature, intense light, high pH, and increased availability of nutrients, especially phosphorous and nitrogen, artificially released by anthropogenic activities [6,7]. These blooms can be hazardous for humans and other animals, and for aquatic organisms, as some cyanobacterial species produce highly toxic secondary metabolites, known as cyanotoxins. Furthermore, recent studies have reported that global climate change can also favor hazardous cyanobacterial species, increasing their growth rate, dominance, persistence, geographical distribution, and activity [8,9].

Many cyanobacteria are found in the form of colonies or filaments that can exert a mechanical influence on the filtration process of zooplankton individuals [10]. Some species or strains of cyanobacteria present toxins that endanger aquatic organisms. Acute effects that may be seen include reduced survival and diminished swimming movements, which may even include complete paralysis [11]. There are also chronic effects, such as reduced fecundity and population growth rate, which may appear as a result of sub-lethal concentrations. However, effects depend on the species, the type of toxin produced, and the concentration used [10]. 

In humans, exposure to cyanotoxins can occur in various ways, however, the oral route is the most important. This is mainly through drinking water, or by eating contaminated foods; it may even involve ingesting water during recreational activities. Furthermore, dermic exposure and inhalation are both possible [6]. Toxic blooms can reappear periodically in hydric environments, and people may be chronically exposed to cyanotoxins at relatively low doses. Known toxins such as microcystins, nodularins, and cylindrospermopsins can cause liver and kidney damage, cytotoxicity, neurotoxicity, skin toxicity, gastrointestinal disturbances, and other problems. These effects may take place within a few minutes of exposure or may appear days later [12]. 

While the main research thrust has been towards investigating toxicity, some studies have shown that cyanobacteria also produce compounds with biological properties that are of pharmaceutical and biotechnological interest. Important pharmacological effects described include anticancer, anti-inflammatory, and antibiotic activities [12]. Cyanobacteria produce an elaborate matrix of secondary metabolites that exhibit a broad range of bioactivities [13] and they are, thus, becoming an exceptional source of important compounds for new medicines [14]. In this context, the objective of the present work is to present a literature review on the evolution of knowledge surrounding toxins produced by cyanobacteria, covering their negative and beneficial impacts. 

## 2. Characterization of Cyanotoxins

The production of cyanotoxins includes risks to human and animal health. Depending on the concentration in the aquatic environment, they can cause severe poisoning, produce chronic diseases such as cancer, and even lead to death. Cyanotoxins are thus an important group of chemical compounds, from the point of view of ecotoxicology, toxicology, and environmental chemistry [9]. 

Cyanotoxins can be divided on the basis of two main criteria: (1) their action mechanism in land vertebrates, especially mammals, in three principal classes: hepatotoxins, neurotoxins, dermatotoxins; and (2) their chemical structure, within which they may be classified as cyclic peptides, alkaloids, or lipopolysaccharides (LPS) [15]. The main cyanobacterial toxins, the genera that produce them, and their mechanism of action are all described in Table 1. 

**Table 1:** Main toxins from cyanobacteria, including genera of main producers and action mechanism.

Cyanotoxins	Genera of main producers	Chemical classification	Action mechanism	References
**Hepatotoxins**				
Microcystins	*Anabaena*, *Planktothrix*, *Nostoc*, *Anabaenopsis*	Cyclic Heptapeptides	Inhibition of protein phosphatases type 1 and 2A	[16,17]
Nodularins	*Nodularia*	Cyclic Pentapeptídes	Inhibition of protein phosphatases type 1 and 2A	[16,17]
Cylindrospermopsins	*Cylindrospermopsis raciborskii*, *Aphanizomenon ovalisporum*, *Aphanizomenon zflos-aquae*	Guanidine alkaloids	Glutathione and protein synthesis as well as cytochrome P450.	[4]
**Neurotoxins**				
Anatoxin-a	*Anabaena*, *Aphanizomenon*, *Planktothrix*	Alkaloid	Irreversible link to the nicotinic receiver S of acetylcholine	[10,18,19]
Anatoxin-a(s)	*Anabaena*	Organophosphate	Irreversible inhibitor of acetylcholinesterase	[19,20]
Saxitoxins	**Dinoflagellates**: *Alexandrium*, *Pyrodinium*, *Gymnodinium* **Cyanobacteria**: *Anabaena circinalis*, *Aphanizomenon sp*., *Aphanizomenon gracile*, *Cylindrospermopsis raciborskii*, *Lyngbya wollei*	Carbamate alkaloids	block sodium channels in nerve axons	[9,16,19]
**Dermatotoxins**				
Lyngbyatoxin-a	*Lyngbya*	Alkaloid	potent tumor promoters, acting through potentiation of protein kinase C (PKC)	[3,6]
Aplysiatoxin	*Lyngbya*, *Schizothrix*, *Planktothrix (Oscillatoria)*	Alkaloids	potent tumor promoters, acting through potentiation of protein kinase C (PKC)	[3,6]
Lipopolysaccharides (LPS)	Cyanobacteria in general	Lipopoly-saccharides	Inflammatory agents, gastrointestinal irritants	[3,21]

Hepatotoxins are cyclic peptides. They either contain five amino acids, in the case of nodularin, which according to Pearson *et al*. [16], are commonly isolated from the filamentous, planktonic cyanobacterium, *Nodularia spumigena*, or seven amino acids, of which the best known are the microcystins, produced by various genera of freshwater cyanobacteria [4], and also in the oceans, isolated from a number of cosmopolitan marine species [22]. In addition, cylindrospermopsin, a cyclic guanidinic alkaloid, is also considered to be a hepatotoxin and is produced by several genera of freshwater cyanobacteria [4]. The mechanisms of cyanobacterial toxicity are diverse, ranging from hepatotoxic, cytotoxics, and neurotoxic effects to the inhibition of protein synthesis [23]. Hepatoxins cause the rupture of the liver structure by hypovolemic shock and an excessive accumulation of blood in the liver. They also inhibit protein phosphatases type 1 or 2 (PP1 or PP2A) within liver cells, essential components in the control of cellular structure and function [24]. As well as working in the liver, hepatotoxins provoke damage to the kidneys, spleen, heart, and other organs [25]. They can also cause long-term effects with chronic exposure, such as promoting tumors, and they have been linked to a higher incidence of cancer in populations that are chronically exposed to microcystins and cylindrospermopsin [10].

Neurotoxins are the most toxic compounds produced by cyanobacteria. They interfere in the neuromuscular system, causing paralysis of the respiratory muscles, and death by respiratory failure in rats after only a few minutes [24]. Neurotoxins of the saxitoxin type are known as PSPs (“paralytic shellfish poisoning”), because their effects were first described in humans poisoned after consuming contaminated bivalve mollusks [11]. They were first isolated from marine dinoflagellates, responsible for the red tide phenomenon. They are highly potent toxins, and many human poisoning cases have been linked to eating marine shellfish contaminated with saxitoxins from dinoflagellates [26]. Many countries have started a substantial monitoring program for saxitoxins in shellfish used for human consumption, as a measure to avoid new cases of poisoning. 

In Brazil, strains of *Cylindrospermopsis raciborskii*, isolated from various regions, have been seen to produce saxitoxins. In the last two decades the occurrence of *C. raciborskii* has been frequent in the water of reservoirs and dams in a number of Brazilian states. In many Brazilian reservoirs, even the newest ones, the genus *Cilindrospermopsis* is already dominant, reaching a number of cells well in excess of the acceptable upper limit for risks to human health [27]. However, no case has been reported so far of human poisoning involving freshwater producers of saxitoxin [26].

Cyanotoxins classified as dermatotoxins, aplysiatoxins and lyngbiatoxins have been found in benthic marine cyanobacteria and are responsible for severe cases of contact dermatitis among those bathing in coastal waters [10].

Another type of toxin produced by many cyanobacteria and also by all gram-negative bacteria is the group of lipopolysaccharides (LPS). These consist of a principal polysaccharide and various secondary ones, among which are long-chain fatty acids and non-phosphate fatty acids [10]. LPS present dematotoxic and inflammatory properties, being recognized as causing fever in mammals. They are involved in toxic shock syndrome, which may worsen the hepatic damage induced by hepatotoxins [28].

## 3. Impacts on Aquatic Ecosystems

Cyanobacteria and their toxins exert effects at all taxonomic levels, including bacteria, algae and plants, but special attention has been given to their effects on aquatic invertebrates, mainly zooplankton [10]. Cyanotoxins can influence the structure of the zooplankton community, especially at times when cyanobacteria are the dominant phytoplankton groups [3]. They can affect *Daphnia pulicaria*, reducing its filtration capacity and survival of offspring [29].

Among cyanotoxins, adverse effects of ingestion of microcystins were observed in various aquatic organisms. Rohrlack *et al*. [30] studied the intestinal absorption and effects of the intake of microcystins on *Daphnia galeata*. They monitored over time the gut structure, blood microcystin concentration, appearance, and movements of *Daphnia* fed *Microcystis* PCC 7806 or a microcystin-deficient PCC 7806 mutant. In all cases, ingestion of the microcystin-producing PCC 7806 wild-type cells resulted in lethal poisoning. Once the microcystin entered the bloodstream and affected neuromuscular communication 5–9 h after feeding, the beat rates of the thoracic legs, mandibles, foregut, and second antennae decreased simultaneously and significantly. The peristaltic movements of the midgut also stopped entirely. Both the wild-type strain PCC 7806 and its mutant type caused loss of cell-cell contact within the digestive epithelium of *Daphnia galeata*, and the intake of 10.2 and 18.3 ng of microcystin per 1 mg body weight caused rapid death of *Daphnia galeata*.

Effects of microcystins on *Daphnia magna* were also studied by Chen *et al*. [31]. They confirmed that in low concentrations of dissolved microcystins there was no poisonous effect on *Daphnia magna*, but at high doses and during long-term exposure the effect was fatal. With long exposure, microcystins began to accumulate in *Daphnia magna* and activity of the enzyme phosphatase was inhibited.

In addition to the microcystins, the effects of saxitoxins have also been studied. The changes in thoracic appendage beat rate in *Daphnia carinata*, exposed to an *Aphanizomenon*, filtrate were evaluated by Haney *et al*. [32]. They demonstrated that the short-term response pattern to the filtrate included an immediate 30%–50% depression of thoracic appendage beat rate. There was also a rise in the post-abdominal rejection rate, followed by slowly decreasing thoracic appendage beat rate, and increasing post-abdominal rejection rate during the 10 min exposure. Nogueira *et al*. [33] found adverse effects of a strain of *Aphanizomenon issastchenkoi* (LMECYA31), producing saxitoxins, on the survival and somatic growth of *Daphnia magna*. Reproduction rates of *Daphnia pulex* and *Moina micrura* evaluated by Costa [34] were negatively affected by a strain of *C*. *raciborskii* producing saxitoxins (T3), but not by another strain (NPLP-1), which does not produce them.

However, as well as daphnids, cianobacteria, algae, diatom and plants can also be affected by cyanotoxins. *Fischerellin*, produced by the cyanobacterium *Fischerella muscicola*, exhibited inhibitory effects on the photosynthesis of other cyanobacteria [28]. Singh *et al*. [35] observed the inhibition of photosynthesis and growth, and an increase in cell lysis in the cyanobacteria *Nostoc muscorum* and *Anabaena*, after exposure to microcystin-LR (MC-LR) for six days. Lipophilic extracts of biomass and culture media of cyanobacterium *Fischerella* sp, strain 52-1, inhibited the growth and photosynthesis and caused ultrastructural damage in green algae *Chlamydomonas* sp. [36]. In addition, cyanobacteria from the brackish water ecosystem of the Baltic Sea have demonstrated negative allelopathic potential towards coexisting phytoplanktonic species. *Anabaena lemmermannii*, *Aphanizomenon flos-aquae* and *Nodularia spumigena* inhibited growth of red algae *Rhodomonas* sp. and the diatom *Thalassiosira weissflogii in vitro* [37]. 

The underwater plant *Ceratophyllum demersum* also presented reduced growth in the presence of 1.0 μg L^−1^ of MC-LR after six weeks, while a concentration of 5 μg L^−1^ of MC-LR reduced the same plant’s growth significantly after three weeks [38]. In a mixed culture experimental approach, *M. aeruginosa* and the duckweed *Lemna japonica* reciprocally inhibited the growth of each other [39].

Although they present greater resistance, mollusks can also be affected by cyanotoxins [10]. Gérard and Le Brient Rouzic [40] evaluated direct exposure to an aqueous solution of MC-LR, which induced a moderate accumulation within the tissues of the gastropod pulmonate *Lymnaea stagnalis*, showing also a strong decrease in egg production in adults. Juhel *et al*. [41] found that *Dreissenia polymorpha* mussels fed with cell suspensions of toxic *Microcystis aeruginosa* CCAP 1450/10 had the lowest rate of food intake, filtration, absorption and fecal loss, and the net energy balance (scope for growth) was also significantly reduced.

Besides the effects of cyanobacteria on aquatic invertebrates, they can also be harmful to fish. In the UK, histopathological investigations of fish deaths during cyanobacterial blooms indicated that the cause of death was mostly due to damage to the gills, digestive tract, and liver. The gill damage was probably caused by the high pH induced by cyanobacterial photosynthesis activity prior to bloom collapse, together with the higher level of ammonia arising from the decomposition of the cyanobacteria. However, gill damage may have enhanced microcystin uptake and thus led to liver necrosis. Other pathological symptoms attributed to toxic cyanobacterial blooms include: damage to the liver, heart, kidney, skin, gills, and spleen [3]. 

Fish, especially phytoplanktivorous species, can be exposed to microcystins while feeding, or by passive means when the toxins pass through the gills during breathing. Fish in the early stages of life are generally more sensitive to toxic compounds than adults and juveniles, probably due to their thin epithelial layer combined with a relatively large body surface area and high metabolic rate. The occurrence of disturbances in the main development processes can often result in death [42]. Liu *et al*. [43] observed that mortality in juvenile loach (*Misguruns mizolepis*) was dependent on the development stage in loach embryos in studies of chronic exposure to microcystin-LR. However, Oberemm *et al*. [44] did not observe apparent acute toxic effects in zebrafish, within the interval of 0.5 to 50 mg/L of microcystin-LR, microcystin-RR (MC-RR), and microcystin-YR (MC-YR). In Table 2, the toxicity of microcystins for some fish species is described.

Palíková *et al*. [45] investigated the toxicity of different blooms of cyanobacteria in the development on embryolarval development of carp *Cyprinus carpio*. Two samples were dominated by *Microcystis* spp., and two others by *Aphanizomenon flos-aquae* or *Planktothrix agardhii*. The results affirmed that both samples of complex cyanobacterial biomass, as aqueous extracts, showed embryotoxic effects such as significant fish mortality, delayed hatching, lower number of hatched embryos, suppression in embryonic development, disturbance of air bladder filling, and significant inhibition of glutathione *S*-transferases (GST). Missing eye pigmentation at 48 h post fertilization and incomplete filling of air bladder after 120 h were observed in some individual carp exposed to higher concentrations of some fractions from all biomasses. Generally higher toxicities were observed in the filament samples dominated by *Aphanizomenon Planktothrix*, while lower toxicities were associated with samples dominated by *Microcystis* spp. 

**Table 2:** Toxicity of microcystins for some species of fish.

Species	Toxic concentrations	Effects	References
Loach (*Misguruns mizolepis)*	0–500 μg MC-LR/L, during 30 days	Mortality and abnormalities such as: pericardial edema and tubular heart, bradycardia, homeostasis, poor yolk resumption, small head, curved body and tail, abnormal hatching.	[43]
Chub (*Leuciscus cephalus*)	0.5, 5, 50 μg MC-LR or MC-RR/L	Dose-dependent decrease of survival rate.	[46]
Zebrafish(*Danio rerio*)	0.5, 5, 50 μg MC-LR or MC-RR/L; 5, 50 μg MC-LR/L	Retarded survival rate and growth. Survival rate reduced by 40%, weight reduced by 25%.	[44]
Medaka (*Oryzias latipes*)	Microinjection MC-LR 1–10 μg /mL (0.1–2 pg or 1–20 pg of toxin injected)	Dose dependent mortality of embryos. Hepatobiliary damages such as hepatobiliary hypertrophy, hepatic hemorrhage and necrosis at late development stages.	[47]
Rainbow trout (*Oncorhynchus mykiss*)	0.5, 5, 50 μg MC-YR or MC-RR/L and 50 μg MC-LR/L	Stimulated hatching	[44]

Tencalla *et al*. [48] investigated the effects of MC-LR on yearling rainbow trout when exposed intraperitoneally or by immersion with algae in water. Concentrations of *M*. *aeruginosa*, 8–16 mg freeze-dried algae/L, were not seen to be toxic for trout when added to the water. However, the trout died within 96 h when 1.440 mg of lyophylized algae/kg of body weight passed through the gills for 18 h. The study showed that the main route taken by microcystins in trout is via the gastrointestinal tract and that the toxicity manifests itself as massive hepatic necrosis.

Oberemm *et al*. [44] studied the effects of microcystins in *Danio rerio*. They demonstrated that embryonic exposure to concentrations of 50 and 5 μg/L MC-LR and MC-RR reduced survival rates by approximately 40% and 20%, respectively. Growth and weight were also reduced by about 25%, with concentrations of 50 and 5 μg/L MC-LR. 

In this context, some scholars have delved into the accumulation of microcystins in the food chain. According to studies conducted by Ibelings *et al*. [49], microcystin levels were especially high in the livers of planktivorous fish, mainly smelt, which puts piscivorous birds at risk. In the same study, microcystins were found in 80% of all zooplankton samples and 89% of all samples of *Dreissena polymorpha*. These authors concluded, therefore, that the transfer of microcystin within the food chain does take place, despite the absence of bio-magnification.

Effects of neurotoxins on fish have also been evaluated. Ossvald *et al*. [50] demonstrated that juvenile three-month-old common carp *Cyprinus carpio* exposed to suspensions of 10^5^ and 10^7^ cell/mL lyophilized cyanobacteria containing anatoxin-a recorded two behavioral changes: rapid opercular movement and abnormal swimming. This interference with fish behavior caused by anatoxin-a may have negative consequences on fish populations due to changes in reproductive and predator-prey interactions. Furthermore, for the highest concentration of cells, all fish died between 24 and 29 h, while for the lowest concentration (10^5^ cell/mL) no deaths were recorded. Oberemm *et al*. [44] tested anatoxin-a in *Danio rerio* and concluded that exposure to 400 μg/L of anatoxin-a altered heart rate at different stages of development. These authors also studied the effects of saxitoxin on *Danio rerio* embryos. When exposed to 500 μg/L saxitoxin, 34% of embryos generated malformations, chiefly lateral and ventral body curvature and edema, and this led to an increased mortality of about 40% during larval development after 21 days.

Ferrão-Filho *et al*. [51] showed that *Cylindrospermosis raciborskii*, producing saxitoxins, altered the swimming activities of *Danio rerio*, decreasing the mean distance covered and mean velocity. Lefebvre *et al*. [52] showed that dissolved saxitoxins caused severe reductions in spontaneous swimming behavior and touch response in larval herring (*Clupea harengus pallasi*). The magnitude of observed deficits was dose dependent, with the greatest degree of impairment occurring at approximately 1 h of exposure. Interestingly, herring larvae at all ages exhibited a significant degree of neurobehavioral recovery within 4–24 h of continuous exposure relative to the 1 h time point.

## 4. Impacts on Human Health

Microcystins have caused human poisoning worldwide. The reported health problems are most likely related to chronic exposure to low microcystin concentrations through consumption of contaminated water and food (agricultural products, fish, prawns, and mollusks), dermal exposure, and inhalation. The liver is the most affected organ in humans, but exposure to the toxin is likely to affect other organs such as kidneys and colon, as evidenced by *in vivo* and *in vitro* studies. As a result, the illnesses attributed to microcystin poisoning are gastroenteritis and related diseases, allergic and irritation reactions, and liver diseases. Some lesions can evolve into tumors and primary liver cancer and colorectal cancers in human populations have been related with microcystin exposure and toxicity [53].

Exposure to cyanobacterial toxins through consumption of contaminated drinking water has caused serious poisoning in humans, even leading to fatalities [18]. Therefore, in 1997 the World Health Organization established a provisional reference value of 1 μg/L for microcystin-LR in drinking water [54]. In addition to the oral route, there is the possibility of the parenteral route of exposure, when surface waters infested by cyanobacteria are used for hemodialysis, considerably increasing the internal dose of toxins, which enter the blood and therefore endanger the life of patients [6]. The first confirmed outbreak of human poisoning involving cyanotoxins took place in the town of Caruaru, Brazil, in 1996, when patients receiving routine renal dialysis treatment began to complain of headache, eye pain, blurred vision, nausea, and vomiting. A total of 76 patients died from liver complications due to the use of water contaminated by cyanotoxins. The analysis confirmed the presence of microcystins and cylindrospermopsin in activated carbon used in the clinic’s water purification system. Microcystins were found in blood and liver of patients [55]. 

Various other cases have been described of human poisoning due to the presence of cyanotoxins dissolved in water. Another episode occurred in Brazil, where the poisoning was attributed to blooms from *Anabaena* and *Microcystis* freshwater genera, producing 2000 reported cases of gastroenteritis and 88 deaths in a period of 42 days [56]. In England, Turner *et al*. [57] reported a case when two recruits on a canoeing exercise drank water from a reservoir containing *Microcystis aeruginosa* and showed left basal pneumonia four to five days later. In addition, in Australia treatment with copper sulfate of cyanbacterial blooms caused cellular lysis, occasioning the hospitalization of 140 children and 10 adults, due to problems in liver and kidneys [58]. In this case, *Cylindrospermopis raciborskii* was identified as the etiological agent [59].

However, beyond the possibility of human contamination through the water supply, there is also the risk of contamination through the food chain. Bioaccumulation of cyanotoxins and transfer through the food chain has been demonstrated in several studies. There is a possibility that these toxins reach humans by the consumption of fish. Some, like tilapia and carp, can consume the cells of cyanobacterial toxins in water, accumulating them in the liver, kidneys, muscles, and viscera [10].

In addition, with respect to neurotoxins, saxitoxins are highly toxic alkaloids that block sodium channels in nerve axons, causing loss of sensation, and paralysis [19]. They comprise a diverse group of toxins and are known as paralytic shellfish toxins, or PST, because their effects were first described in humans poisoned after eating contaminated shellfish [10]. On a global scale, approximately 2000 cases of human poisoning are reported annually, with 15% mortality due to consumption of fish or shellfish that fed on marine dinoflagellate producers of saxitoxins. The most common symptoms of human poisoning by saxitoxins include dizziness, numbness of the mouth and extremities, muscle weakness, nausea, vomiting, thirst, and tachycardia. Symptoms may begin 5 min after ingestion and death may occur from 2 to 12 h [60].

Some descriptions of allergic reactions due to cyanobacteria have also been published [6]. Exposure to LPS endotoxins has been associated with a series of pathological effects in humans, such as gastrointestinal diseases, allergic reactions, and cutaneous or ocular irritation. These are indirect effects, with LPS setting off a cascade of host-mediated responses. Initially, the monocytes and macrophages are stimulated, and next the neutrophils and platelets join in microcapillaries, causing vascular damage. Inflammatory cells release a variety of endogenous mediators, including arachidonic acid metabolites, platelet activation factor, cytokines, nitric oxide, toxic O_2_ metabolites, vasoactive amines, proteases and products of the complement and coagulation cascades [61]. 

Among the different types of gram-negative bacteria, the lipid A moiety is considered the LPS component that is responsible for toxic effects; it can be extremely variable and even completely inactive. As the lipid A subunit structure in the molecule of the LPS cyanobacterium has not been clearly identified, no definitive conclusion should be drawn about its degree of toxic potential, although some indirect data suggest that LPSs are implicated in poisoning. Some tests have indicated that different extracts of cyanobacteria can induce a high degree of skin sensitivity, independent of the production of intracellular cyanotoxins, suggesting a possible role for other constituents of cyanobacteria, among them LPS endotoxins. On the other hand, during cyanobacterial blooms, other organic compounds are dissolved in water, such as aldehydes, terpenes, and ketones, and some of them present irritating and sensitizing properties. However, it cannot be discarded that in these studies such effects on the skin and mucous membranes may be due to the concomitant presence of different etiologic agents [6].

In addition to the acute effects caused by cyanotoxins, chronic exposure to low doses of these can lead to tumor promotion and cancer development in humans. Cancer is considered a major public health problem in both developed and developing countries and is among the leading causes of mortality worldwide [62,63]. It is increasing with changes in lifestyles and nutrition and with global warming [64]. According to an estimate by the American Cancer Society, 7.6 million people died from cancer worldwide during 2007 [63].

Recent studies have indicated that many health problems, both acute and chronic, are probably related to exposure to low concentrations of microcystins, present in the water used for consumption [65]. The revelation that cyanobacterial hepatotoxins cause protein phosphatase inhibition has raised the disturbing possibility that human exposure to non-lethal doses of these compounds may contribute to the development of cancer [16]. Recently, the International Agency for Research on Cancer (IARC) has classified microcystin-LR as a possible human carcinogen (Group 2B) [66].

Epidemiological studies have posited an association between the increased incidence of liver and colon cancer and drinking water sources that were potentially contaminated with microcystins in certain areas of China. This country’s rate of human hepatocellular carcinoma is one of the highest in the world and varies geographically. In addition to the proven risk factors that correlate with the increased incidence of the disease, such as the intake of aflatoxin (AFB1) from foods and infection by hepatitis B virus, the third significant element of association is the source of drinking water. The highest cancer mortality rates are observed in regions where sources of drinking water are ponds and ditches, compared to lower incidence in regions where drinking water is drawn from deep wells [66]. Data obtained by Ueno *et al*. [67] support the hypothesis that the blue-green algal MC toxin in the drinking water of ponds, ditches and rivers, or both, is one of the factors involved in the high incidence of primary liver cancer (PLC) in China. 

Another tumor strongly related to ingestion of contaminated water with microcystin is colorectal cancer, which is increasing rapidly in many countries. Recent studies in China show that consumption of water with more than 50 pg/mL microcystins multiplies the risk of colorectal cancer by 7.9 [68].

The main mechanism associated with the potential carcinogenic activity of cyanotoxins is through specific inhibition of serine/threonine phosphatases (PP2A and PP1). This results in hyperphosphorylation of proteins, and thereby disrupts many cellular processes and alters cytoskeletal structures. In addition to protein phosphatase inhibition, MCs can cause oxidative stress by increasing the formation of reactive oxygen species (ROS), modifying intracellular antioxidant enzymes. Oxidative stress and mitochondrial alterations play a critical role in microcystin-induced apoptosis, as well as in DNA damage, two mechanisms implicated in tumor initiation. Microcystins are considered to be liver-specific because they enter cells through the multispecific transport system for organic anions and bile acids. The predisposition for hepatic damage from microcystins thus probably depends on the high concentration of these transporters in the hepatocyte membrane. However, organic anion transporters are not only expressed in the liver, but also in the gastrointestinal tract, kidneys, and brain, as components of the blood-brain barrier [69]. 

Other studies have also revealed that microcystin-LR induced the oxidation of nitrogenous bases, the pyrimidines and purines. These results indicate that oxidative stress is an important mediator of microcystin-LR-induced hepatotoxicity and that reactive oxygen species (ROS) are also involved in microcystin-LR-induced genotoxicity. Free radical attack on DNA generates different types of DNA damage-modified DNA bases, DNA strand breaks, DNA-protein cross-links, and abasic sites [70].

Žegura *et al*. [70] have explored the role of ROS in microcystin-LR-induced DNA damage, using cultured cells derived from human hepatoma (HepG2). They found that, even after 12 h of exposure to microcystin-LR, the oxidized purines were not repaired. This indicates that microcystin-LR-induced formation of oxidized purines is faster than their removal by cellular DNA repair mechanisms, leading to accumulation of these lesions.

The effect of non-cytotoxic concentrations of microcystin-LR were investigated by Žegura *et al*. [69], examining generation of ROS and DNA damage in cell lines derived from human colon adenocarcinoma (Caco-2), human astrocytoma (IPDDC-A2), and human B-lymphoblastoid (NCNC) cultured in the laboratory. They observed that intracellular ROS production increased in Caco-2 and IPDDC-2A cells but not in NCNC. The main finding of the study was the relatively high sensitivity of the colon cells (Caco-2) with respect to genotoxicity of microcystin-LR. In Caco-2, microcystin-LR increased DNA strand breaks, and a significant increase in DNA damage was observed after 2 h incubation at the highest microcystin-LR concentration (5 μg/mL). Longer exposure of Caco-2 cells at non-cytotoxic doses (0.2, 1 and 5.0 μg/mL) of microcystin-LR induced intracellular ROS, as well as breaks in DNA. However, Lankoff *et al*. [71] found a reduced mitotic index in stimulated human lymphocytes exposed to more than 10 μg/mL microcystins-LR for 24 h.

## 5. Anticancer Activity of Cyanobacteria

As shown above, cyanobacterial cyclopeptides, including microcystins and nodularins, are considered a danger to human health, because of the possible toxic effects of high consumption [72]. However, from the pharmacological standpoint, microcystins are stable hydrophilic cyclic heptapeptides with the potential to cause cellular damage following uptake via organic anion transporting polypeptides (OATPs) [73]. Their biological effects involve intracellular inhibition of the catalytic subunit of protein phosphatase 1 (PP1) and PP2, glutathione depletion and generation of reactive oxygen species (ROS) [74]. Interestingly, certain OATPs are prominently expressed in cancers as compared with normal tissues, suggesting that microcystins may be potential candidates for the development of anticancer drugs [73]. It should be noted that cancer cells live in a state of increased basal oxidative stress, which makes them vulnerable to further ROS insults induced by exogenous agents. Therefore, microcystin analogues can selectively kill cancer cells that express (AOTP) without causing significant toxicity to normal cells by exploiting the differences between normal and cancer cells redox [75]. Cyanotoxins include a rich source of natural cytotoxic compounds with the potential to target cancers by expressing specific uptake transporters. Their structure offers opportunities of combinatorial engineering to improve the therapeutic index and resolve organ-specific toxicity issues [73]. 

The drugs currently used to treat cancer cause many unwanted side effects and, increasingly, natural products from medicinal plants have gained significance in the treatment of this disease. Natural products and their derivatives represent more than 50% of all drugs in clinical use in the world and almost 60% of drugs approved for cancer treatment are of natural origin [64]. The ability of natural products to inhibit the growth of cancer cell lines may lead to the discovery of effective anticancer drugs.

Cyanobacteria are considered a promising source of novel pharmaceutical lead compounds, and a large number of chemically diverse metabolites have been obtained from them [76]. *In vitro* studies of soblidotin, a synthetic analog of dolastatin 10, showed promising results against human colon adenocarcinomas and this compound has progressed to phase II clinical trials. Synthadotin, derived from dolastatin 15, has shown promising results in phase II clinical trials of inoperable, locally advanced or metastatic melanoma [77]. Cryptophycin 1 was isolated in the laboratory from *Nostoc* sp. GSV224, and showed action on nasopharyngeal cancer cells and human colorectal cancer cells, being found to be 100–1000 times more potent than the anticancer drugs currently available, such as taxol or vimblastine [63]. Anticancer properties of the cyanobacteria *Oscillatoria* spp. were studied by Nair and Bhimba [78], and the results clearly indicated that *Oscillatoria boryana* showed anticancer activity against cell lines of human breast cancer. 

Cyanobacteria of pharmacological and toxicological significance include the genera *Anabaena*, *Oscillatoria*, *Microcystis*, *Nodularia*, *Cylindrospermopsis*, and *Lyngbya*. *Lyngbya* spp. and *Microcystis* spp. are readily collected and cultured in the laboratory for the isolation of compounds in the mg range [79]. It is of note that cyanobacteria also belong to promising groups of diverse taxa of marine organisms with pharmacological importance, such as *Lyngbya*, *Oscillatoria*, *Symploca*, *Calothrix*, *Leptolyngbya*, *Dichothrix*, *Geitlerinema*, *Schizothrix*, *Aphanothece*, *Blennothrix*, and *Synechocystis*. These produce diverse structural classes of metabolites such as acyclic peptides, linear decadepsipeptides, linear lipopeptides, linear alkynoic lipopeptides, cyclic depsipeptides, cyclic undecapeptides, cyclic hexapeptides, lipophylic cyclic peptides, paracyclophanes, sesterterpenes, glycolipids, polyphenolic ethers, and macrolactones, among others [80].

*Lyngbya* is a common and accessible genus of cyanobacteria, which is distributed worldwide throughout tropical and subtropical regions. Its filaments often form a wide layer or mats of varied thickness and then produce large benthic or surface blooms in freshwater and seawater. There are an increasing number of species of this genus that have been found to produce an impressive variety of structurally diverse compounds with a range of biological activities [81]. Currently, the most important species of the genus *Lyngbya* in terms of production of secondary metabolites are: *L*. *majuscula*, *L*. *martensiana*, *L*. *aestuarii*, and *L*. *wollei*. Substructures containing peptides represent an important group of secondary metabolites from cyanobacteria. Most biological activities reported for the compounds obtained from *Lyngbya* fall into two main categories: cytotoxicity or protease inhibition [77].

*Lyngbya majuscula* collected from various coastal and deep-sea regions has proved to be a predominant source, among other cyanobacterial genera, for the production of various chemical classes of natural products with anti-proliferative, anti-tumor and anti-cancer properties, regardless of their geographical variation [80]. More than 50% of marine cyanobacteria are potentially exploitable for the extraction of bioactive substances that are effective in destroying cancer cells by inducing apoptotic death, or affecting cell signaling through activation of members of the protein kinase-C (PKC) family signaling enzymes [64]. The most studied species of marine cyanobacteria include: *Nostoc*, *Calothrix*, *Lyngbya* and *Symploca*. However, the most cytotoxic compounds have been isolated from the benthic marine cyanobacteria of *Lyngbya* spp. and *Symploca* spp. [82].

Efforts to extract drugs from the sea started in the late 1960s. From 1977 to 1987, about 2500 new metabolites were reported from a variety of marine organisms. These studies clearly demonstrated that the marine environment is an excellent source of new chemicals, not found in terrestrial sources. More than 10,000 compounds have been isolated from marine organisms and about 300 patents on bioative marine natural products were issued between 1969 and 1999 [83]. Pioneering studies by Richard E. Moore, of the University of Hawaii, in the mid-1970s revealed that cyanobacteria are an extremely rich source of secondary metabolites. Many of these compounds uniquely combine structural features of peptides with lipid sections, and then become further functionalized with unusual oxidation, methylation, and halogenations. Moreover, these preliminary studies showed that many were highly bioactive, displaying activity in cancer models, toxicity to macroscopic life forms, or anti-infective activity [84]. 

One of the reasons why marine cyanobacteria have evolved this ability to produce such extensive bioactive molecules is because they are prokaryotes that have developed beyond a microscopic lifestyle, and therefore require an arsenal of chemical defenses to ward off predation by various types of macrograzers [85]. An emerging trend in the pharmacology of marine cyanobacterial cytotoxins is that they target hypoxia-inducible factor-1 (HIF-1) and processes downstream of mitochondrial respiration [86]. In this context, the capacity of cyanobacteria to produce an abundant amount of toxic metabolites may simply result from the ways in which the extracts and compounds indicated were evaluated in biological terms [84].

Oftedal *et al*. [82] investigated the potential anticancer lineage of marine benthic cyanobacteria on the shores of the Baltic Sea and found that extracts from *Anabaena* sp. M27, M30 and M44 rapidly induce apoptosis in cells from acute myeloid leukemia (AML) in humans, compared to a higher-than-therapeutic concentration of daunorubicin in these cells. The high potency and rapid action of M27, M30 and M44 against leukemia cells is advantageous for *in vivo* therapy, as it involves a short burst of drug, allowing less time for the formation of potentially harmful metabolites of drugs generally. These same authors also found that the combination of a moderate concentration of the anticancer drug daunorubicin with extracts from *Anabaena* spp. M27, M30, or M44 induced a synergistic apoptotic response in AML cells of rats. Moreover, the extract showed M44 appeared to protect the cardiac cells against toxicity induced by daunorubicin. However, new strategies for the treatment of AML should be composed of different drugs to cover a broader range of cell death pathways, since acquired apoptotic resistance caused by deregulation of death-signaling mechanisms is a common feature in malignant cells [87,88]. If two compounds act synergistically, their combination may allow lower doses of each, and thus lower toxicity, compared with monotherapy [82].

The main compounds with anticancer activity can be inserted into three groups divided according to their mechanisms of action: dolastatins and its analogs, protease inhibitors and cell cycle targeting compounds. 

### 5.1. Dolastatins and Its Analogs

One of the prime examples of potent disruptors of microtubules arising from marine cyanobacteria is the dolastatins, particularly dolastatins 10 and 15. Dolastatins were originally reported from the Indian Ocean sea hare, *Dolabella auricularia*. In the 1990s, dolastatin 10 started phase I of clinical trials and progressed to phase II, but the tests were then stopped because of disappointing results [89]. Based on the structural model of dolastatin 10, various analogs were synthesized, which resulted in the development of a simplified derivate of dolastatin 10, TZT-1027, an agent that depolymerizes microtubules, with antivascular activity which disrupts newly formed tumor vasculature. TZT-1027 showed antiangiogenic activities in *in vivo* tests in a chorioallantoic membrane embryo (CAM), and in an *in vitro* tube formation assay on human umbilical vein endothelial cells (HUVEC) [90]. However, in another phase II study, carried out in patients with advanced non-small-cell lung cancer after treatment with platinum-based chemotherapy, TZT-1027 did not show anti-cancer activity [91]. 

As well as dolastatin 10, dolastatin 15 is another peptide from cyanobacteria originally reported from the sea hare *D*. *auricularia*, but it has not started clinical trials. Despite this, two synthetic analogs of dolastatin 15, compounds LU-103793 and ILX651, were developed as candidates for clinical testing. Phase I trials with ILX651, in patients with advanced solid tumors, indicated that the compound is well tolerated and no cardiotoxicities were observed, as observed with the compound LU-103793. Compound ILX651 has completed at least three rounds of clinical tests II in patients with hormone refractory prostate cancer and advanced or metastatic non-small-lung carcinoma [89].

### 5.2. Proteases Inhibitors

Various cyclic depsipeptides derived from freshwater and marine cyanobacteria exhibit powerful inhibition of serine proteases, such as elastase, chymotrypsin, and trypsin. This class of enzymes is involved in various disease states, including the destruction of the extracellular matrix of the articular cartilage and bones in arthritic articulation, emphysema, gingivitis and infections. Thus, the inhibition of proteases would represent new therapeutic targets in the treatment of cancer. Among the first examples of serine proteases inhibitors arising from marine filamentous cyanobacteria are the lyngbyastatins 4, 5, 6, and 7. These were isolated from marine cyanobacteria in Florida, belonging to the genus *Lyngbya*. During *in vitro* studies, it was confirmed that they are potent elastase inhibitors with IC_50_ values in the nanomolar range. Another compound, somamide B, re-isolated from the Florida strain, *Lyngbya* sp., is also capable of inhibiting elastase. The investigation of a strain of *Symploca* sp. from Papua New Guinea resulted in the compound symplocamide A, which was found to inhibit selectively chymotrypsin with an IC_50_ of 0.38 uM; it is also cytotoxic for non-small lung NCI-H460 cells and neuroblastome cells with values of IC_50_ of 40 and 29 nM [89].

### 5.3. Cell Cycle Targeting Compounds

The cell cycle is a delicate mechanism, comprising cell growth and its division into two daughter cells. Some substances which are able to disrupt the normal functioning of the cell compromise the viability of this mechanism, a result that can be directly related to apoptosis. A typical cell injury induced by marine cyanobacterial compounds is the disruption of microtubules and active proteins. As these proteins are directly involved in mitosis, changes occur in the normal functioning of the cell cycle. The result is, most frequently, interruption of G2/M phase arrest [89]. The compound calothrixin A, an indolophenanthridine isolated from *Calothrix*, is an example of a bioactive metabolite that in different human cancer cell lines induced arrest of the cell cycle in its G2/M phase. However, calothrixin A, as well as causing an arrest in G2/M phase in a leukemia cell line at 1 μM and 10 Μm, was also responsible for a cumulative arrest in S phase [92]. 

The apratoxin compounds are a class of powerful cyclic depsipeptides with cytotoxic action isolated from various strains of *Lyngbya* sp., from Guam, Palau, and Papua New Guinea [89]. Apratoxins are highly cytotoxic metabolites with a new skeleton, having both peptides and polycetide fragments [80]. Within this class there is the compound apratoxin A, isolated from the marine cyanobacterium *Lyngbya majuscula*, which exhibited cytotoxic effects *in vitro* in various human tumor cell lines, with IC_50_ values varying from 0.36 nM in colon LoVo carcinoma cells to 0.52 nM in epidermal KB carcinoma cells. However, *in vivo*, it was only marginally active against a colon tumor and was not effective against a breast tumor [13]. In gene expression profiling and DNA content analysis, the natural product, apratoxin A, showed significant inhibiting activity on cell division, keeping the cell cycle in G1 phase and inducing apoptosis. By the functional genome method, apratoxin A was seen to block the fibroblast growth factor receptor (FGFR), preventing phosphorylation and activation of the STAT3, which is a downstream effector of the FGFR signaling pathway. Fibroblast growth factor (FGF) plays a key role in angiogenesis and cell proliferation. Inactivation of STAT3, in turn, antagonizes FGF signaling and starts apoptosis cascade [93]. Based on proteomics studies, it has also been shown that apratoxin A prevents the *N*-glycosylation of endoplasmic reticulum receptors, resulting in depletion of cancer-associated receptor tyrosine kinases in cancer cells [89]. 

A similar mechanism to that of apratoxin A has been observed in the potent antiproliferative cyclic depsipeptide coibamide A, isolated from the Panamanian marine cyanobacterium, *Leptolyngbya* sp. This compound has presented powerful cytotoxicity against human lung cancer in cell line NCI-H460 [94]. Furthermore, the compound Lyngbyabellin B, isolated from *Lyngbya majuscula*, exhibits effects on human Burkitt lymphoma cells [64].

Other compounds, such as curacins, have been explored for their antimytotic activity [89]. Among these, curacin-A stands out, isolated from tropical marine benthic cyanobacterium *Lyngbya majuscula* from Curaçao, and it is in pre-clinical trials for cancer treatment. The compound is exceptionally potent as an anti-proliferative and cytotoxic agent, as shown in its capacity to block cell cycle progression by interacting with the colchicine binding site in tubulin and inhibiting microtubule polymerization [64]. It also displays inhibitory activity selectively on colon, renal, and breast cancer-derived cell lines. Simultaneously, there has been considerable interest in producing curacin A by total synthesis and producing synthetics to explore structure-activity relationships in this potential new drug class [95]. 

## 6. Conclusions

Cyanobacteria are important in different ecosystems, but in favorable conditions they can dominate aquatic environments and proliferate unduly. Human activities are mainly responsible for the large supply of nutrients in water bodies, contributing to an increased intensity of cyanobacterial blooms. 

Cyanotoxins are responsible for severe poisoning in aquatic organisms and humans. Ingesting cells of *Microcystis* generates adverse effects in various aquatic organisms. In the case of *Dapnhia galeata* fed with the wild type *Microcystis aeruginosa* all cases produced lethal poisoning. As regards fish, initial life phases were more sensitive to microcystins, because the toxic effects caused disturbances in the main development processes. Anatoxins also generated behavioral alterations such as rapid opercular movement and abnormal swimming in juvenile carp *Cyprinus carpio*. Saxitoxins produced lateral malformations in embryo of *Danio rerio* and increased mortality.

In humans, poisoning by ingestion of contaminated water with cyanotoxins has even led to fatalities. Furthermore, increased incidence of liver and colon cancer has been associated with the presence of cyanotoxins in drinking water sources. Microcystins cause oxidative stress and mitochondrial changes, two mechanisms involved in tumor initiation.

Although cyanotoxins can cause damage, they are also considered a rich source of natural cytotoxic compounds with potential anticancer action. Marine cyanobacteria have evolved and distinguished themselves for their ability to produce bioactive molecules, thanks to their status as prokaryotes that have developed beyond a microscopic lifestyle demanding an arsenal of chemical defenses to ward off predation by various types of macrograzers. Due to the cytotoxicity of these bioactive molecules in various tumor cell lines, several different compounds produced by cyanobacteria have emerged as a model for the development of new anticancer drugs. Among the various compounds with anticancer activity, Curacin A stands out as a potent antiproliferative and cytotoxic agent, and it is in pre-clinical trials for treatment of cancer. Apratoxin A was seen to have significant inhibitory activity on cell division, keeping the cell cycle in the G1 phase and inducing apoptosis. Compound ILX651 has already finished three rounds of clinical trials II in patients with hormone refractory prostate cancer and advanced or metastatic non-small-lung carcinoma.

However, given the great potential of cyanobacteria for the production of compounds with importance in the discovery of new medicines, there is also the need for further efforts to identify biomolecules, develop new methods to isolate cyanobacterial toxins and conduct new research on the compounds that may be obtained from cyanobacteria with anticancer activity.
